# GRIM-19-mediated induction of mitochondrial STAT3 alleviates systemic sclerosis by inhibiting fibrosis and Th2/Th17 cells

**DOI:** 10.1038/s12276-024-01366-0

**Published:** 2024-12-06

**Authors:** Ha Yeon Jeong, Jin-Sil Park, Jeong Won Choi, Kun Hee Lee, Seung Cheon Yang, Hye Yeon Kang, Sang Hee Cho, Seon-Yeong Lee, A Ram Lee, Youngjae Park, Sung-Hwan Park, Mi-La Cho

**Affiliations:** 1 Lab of Translational ImmunoMedicine, Catholic Research Institute of Medical Science, College of Medicine, The Catholic University of Korea, Seoul, 06591 South Korea; 2 Department of Pathology, College of Medicine, The Catholic University of Korea, Seoul, 06591 South Korea; 3 The Rheumatism Research Center, Catholic Research Institute of Medical Science, College of Medicine, The Catholic University of Korea, Seoul, 06591 South Korea; 4 Department of Medical Sciences, Graduate School of The Catholic University of Korea, Seoul, 06591 South Korea; 5 Division of Rheumatology, Department of Internal Medicine, Seoul St. Mary’s Hospital, College of Medicine, The Catholic University of Korea, Seoul, 06591 South Korea

**Keywords:** Autoimmunity, Mitophagy

## Abstract

The gene associated with the retinoid–IFN-induced mortality-19 (GRIM-19) protein is a regulator of a cell death regulatory protein that inhibits STAT3, which is a critical transcription factor for interleukin (IL)-17-producing T (Th17) cells and a key integrator of extracellular matrix accumulation in systemic sclerosis (SSc). This protein is also a component of mitochondrial complex I, where it directly binds to STAT3 and recruits STAT3 to the mitochondria via the mitochondrial importer Tom20. In this study, the role of GRIM19 and its relationship with STAT3 in SSc development was investigated using a murine model of SSc. We observed a decrease in the level of GRIM-19 in the lesional skin of mice with bleomycin-induced SSc, which was negatively correlated with the level of STAT3. Overexpression of GRIM-19 reduced dermal thickness and fibrosis and the frequency of Th2 and Th17 cells in SSc mice. Mitophagic dysfunction promoted fibrosis in mice lacking PINK1, which is a mitophagy inducer. In an in vitro system, the overexpression of GRIM-19 increased the level of mitochondrial STAT3 (mitoSTAT3), induced mitophagy, and alleviated fibrosis progression. MitoSTAT3 overexpression hindered the development of bleomycin-induced SSc by reducing fibrosis. These results suggest that GRIM-19 is an effective therapeutic target for alleviating the development of SSc by increasing mitophagy.

## GRIM-19 regulates STAT3 to combat systemic sclerosis progression

Systemic sclerosis is an autoimmune disease causing skin and organ fibrosis. The exact cause is unknown, but inflammation plays a key role. Researchers found a gap in understanding how the GRIM-19 protein affects SSc. Ha Yeon Jeong and colleagues conducted experiments on mice to explore this. The study involved injecting mice with a substance to induce SSc and then treating them with a GRIM-19 plasmid (a small DNA molecule). This experiment aimed to see if GRIM-19 could reduce fibrosis. The study type was an experiment involving 8-week-old male mice. Results showed that overexpression of GRIM-19 reduced skin thickness and inflammation in SSc mice. The researchers concluded that GRIM-19 helps control fibrosis by interacting with mitochondrial STAT3 (a protein involved in cell signaling). Future research could explore GRIM-19 as a potential treatment for SSc.

This summary was initially drafted using artificial intelligence, then revised and fact-checked by the author.

## Introduction

Systemic sclerosis (SSc) is an autoimmune disease characterized by fibrosis, angiopathy, and the production of inflammatory cytokines^1,2^. The etiology remains unclear, but SSc is initiated by inflammation that is induced following vascular injury^3^. Typically, immune responses are activated to repair damage with increases in the levels of inflammatory cytokines including transforming growth factor (TGF-β), interleukin (IL)-6, and tumor necrosis factor-α. This occurs in damaged tissue and triggers the differentiation of fibroblasts into myofibroblasts as a key step in wound healing^4–6^. Chronic inflammation induces continual myofibroblast activation, which is associated with the excess production of extracellular matrix components and leads to the induction of fibrosis and impairing cell function^7–9^.

TGF-β is a key driver of fibrosis, and TGF-β/SMAD signaling directly activates STAT3^4,10^. The level of STAT3 phosphorylated at tyrosine 705 is elevated in the fibrotic skin of SSc patients with signals triggered by profibrotic kinases, such as JAK2 and JNK, and contributing to TGF-β-mediated fibroblast STAT3 activity^10,11^. STAT3 inactivation induces antifibrotic effects including the inhibition of TGF-β-induced myofibroblast differentiation and collagen release^11^. A recent study implicated the metabolic conditions attributable to the accumulation of damaged mitochondria in the development of SSc^12^. As a transcription factor, STAT3 is active in the nucleus but is also present in the mitochondria where it plays a key role in the activation of complexes I and II and the electron transport chain (ETC) while also regulating the generation of reactive oxygen species (ROS)^13,14^^.^ The overexpression of mitochondrial STAT3 (mitoSTAT3) inhibits ischemia-mediated ETC changes and ROS production^15^. To date, whether mitoSTAT3 is involved in SSc or fibrosis is unknown.

The GRIM-19 (genes associated with retinoid–IFN-induced mortality-19) protein was originally identified as a nuclear protein that augments apoptotic death induced by interferon (IFN)-β and all-trans retinoic acid^16^. Subsequent studies reported that GRIM-19 impairs STAT3-dependent transcription by directly binding to STAT3^17^ and improves acute graft-versus-host disease and rheumatoid arthritis by downregulating STAT3^18,19^. It has also been shown to be essential for mitochondrial complex I assembly and function^20^ by acting as a chaperone of STAT3 through the direct binding of STAT3 and subsequent mitochondrial recruitment via the mitochondrial importer Tom20^17,21,22^. However, it is unknown whether GRIM-19 contributes to the development of SSc. Thus, in this study, the effects of GRIM-19 on the development of SSc, specifically those arising from its interaction with STAT3, were investigated. Our results suggest that GRIM-19 controls fibrosis through a mechanism involving its relationship with mitoSTAT3.

## Materials and methods

### Animals

Eight-week-old male C57BL/6 mice were purchased from Orient Bio, Inc. (Seongnam, Korea). PINK1 knockout mice were provided by Dr. Eunhye Joe (Ajou University School of Medicine, Korea). Animals were maintained under specific-pathogen-free conditions at the Institute of Medical Science of the Catholic University (Korea) and were fed standard mouse chow and water. To establish a murine SSc model, mice were subcutaneously injected with 50 μg of bleomycin (#HY-17565A, MedChemExpress) dissolved in 100 μL of PBS (#CBP3071, Dynebio) daily for 2 weeks. In parallel with the start of bleomycin treatment, the mice were injected intravascularly once a week for 5 weeks with 200 µg of pFLAG-CMV-5a R12 mouse (m) GRIM-19 plasmid, pEG-mitochondrial localization signal (MLS)-mSTAT3-FLAG plasmid, or mock vector prepared in 1 mL of saline (#GIB-21600–069, Gibco). On Day 35 after the start of treatment, the mice were euthanized, and their skin tissues were analyzed histologically as described below. All experimental procedures were approved by the Animal Research Ethics Committee of the Catholic University of Korea and conformed with the United States National Institutes of Health guidelines (permit no. CUMC-2022-0076-02, CUMC-2023-0048-01).

### Plasmid construction

DNAs encoding human (h) GRIM-19 and mouse (m) GRIM-19 were inserted into the pFLAG-CMV-5a vector as well as DNA encoding GRIM-19 (accession no. NM_015965.6, NM_023312.3). The gene encoding MLS-mSTAT3 was inserted into the pEGFP-N3 vector, and the resulting MLS-mSTAT3 protein was fused to the FLAG tag. The human/mouse STAT3^Y705F^ Flag pRc/CMV-encoding plasmid (STAT3^Y705F^; tyrosine-705 replaced by phenylalanine) was a gift from Dr. Jim Darnell (plasmid #8709; Addgene).

### Histology

Skin tissues of the mice were fixed overnight in 10% (v/v) neutral-buffered formalin (#HT501320, Sigma), embedded in paraffin, and sectioned (5 µm thick). Dermal and epidermal thickness and collagen accumulation were scored via sections stained with hematoxylin and eosin (H&E) and Masson’s trichrome (MT). In addition, skin sections were immunohistochemically analyzed via primary antibodies against α-SMA (#ab7817, Abcam, 1:1000), Col1α1 (#PA5–29569, Invitrogen, 1:500), GRIM-19 (#ab110240, Abcam, 1:500), STAT3 (#9139, Cell Signaling, 1:600), TGF β1 (#BS-0086R, Bioss, 1:100), IL-6 (#NB600–1131; Novus, 1:400), IL-17A (#ab79056; Abcam, 1:200) and anti-IL-1β (#NB600–633; Novus, 1:200). After 2 h of incubation at room temperature with the above antibodies, the sections were incubated for 30 min with a horseradish peroxidase-conjugated secondary antibody. The final colored products were developed via chromogen diaminobenzidine. All sections were examined under a photomicroscope (Olympus). Areas positive for fibrosis, as observed on the MT-stained sections, were analyzed via ImageJ software, and the number of antibody-positive cells was visually enumerated.

### Cell culture and transfection

NIH3T3 and human embryonic kidney 293 (HEK293) cells were cultured to 70–80% confluence in DMEM supplemented with 10% fetal bovine serum (FBS, #12800017 and #16000044, respectively, Thermo Fisher Scientific [Thermo]) and 100 U penicillin/streptomycin/mL (#15140–122, Gibco) in a humidified incubator at 37 °C under 5% CO_2_. Cells were transfected with the plasmids via X-tremeGENE HP DNA transfection reagent (#6366236001, Roche) according to the manufacturer’s recommendations. Culture medium was replaced with fresh DMEM supplemented with 10% FBS and 2 µg or 10 µg of plasmid was added. After 16–18 h, cells were washed with PBS and starved for 12 h in DMEM supplemented with 1× insulin-transferrin-selenium A solution (#GIB-51300–044, Gibco). Transfected cells were cultured with recombinant human IL-6 (#206-IL-050, R&D) (10 ng/mL) for 12 h or 48 h.

### Western blotting

Whole-cell lysates or mitochondrial fractions were prepared from HEK293 cells via RIPA protein lysis buffer (#89901, Thermo) containing the Halt phosphatase inhibitor (#78441, Thermo), which is a proteinase inhibitor, and EDTA (#78438, Thermo). Mitochondria were isolated from cultured cells via a dedicated kit (#89874, Thermo) following the manufacturer’s instructions. Protein concentrations were determined via a Bradford protein assay (#23225, Thermo). Proteins were separated via SDS‒PAGE, transferred to Hybond enhanced chemiluminescence (ECL) membranes (#10600001, Cytiva), and incubated with antibodies against GRIM-19 (#ab186848, Abcam, 1:1000), phosphorylated (p)-STAT3 (Tyr705) (#9145 L, Cell Signaling, 1:1000), p727 STAT3 (Ser727) (#9134 L, Cell Signaling, 1:500), STAT3 (#9139, Cell Signaling, 1:1000), LC3B (#ab48394, Abcam, 1:1000), COX IV (#ab33985, Abcam, 1:1000), α-SMA (#ab7817, Abcam, 1:1000), connective tissue growth factor (CTGF) (#ab6992, Abcam, 1:1000), or β-actin (#sc-47778, Santa Cruz, 1:1000). Protein bands were visualized via the SNAP i.d. protein detection system (Millipore) and X-ray film (EA8EC, Agfa).

### Cell staining for flow cytometry

Cells isolated from the peripheral blood of mice were stimulated for 4 h with phorbol myristate acetate (#p8139, Sigma‒Aldrich) (25 ng/mL) and ionomycin (#I0634, Sigma‒Aldrich) (250 ng/mL) in the presence of GolgiStop (#55474, BD Biosciences). Then, they were stained with a cell-surface-binding PerCP5.5-conjugated anti-CD4 antibody (#45–0042–82, Invitrogen, 1:100), permeabilized with Cytofix/Cytoperm solution (#55474, BD Biosciences) and stained with PE-conjugated anti-IL-4 (#554435, BD Biosciences, 1:100) and FITC-conjugated anti-IL-17 (#11–7177–81, eBioscience, 1:100). Flow cytometry analysis of stained cells was performed on a CytoFLEX flow cytometer (Beckman Coulter). Data were analyzed via FlowJo software (Tree Star).

### Confocal laser scanning microscopy

Paraffin-embedded tissue sections (5 µm thick) were incubated overnight at 4 °C in 10% (v/v) normal goat serum (NGS) (#S-1000, vector), 0.1% (v/v) Tween 20 (#P1379–500 ML, Sigma) or the following primary antibodies in PBS: anti-CD4 (#NBP2-25191, Novus, 1:200), anti-IL-4 (#PA5–25165, Invitrogen, 1:200), and anti-IL-17 (#11-7177-81, eBioscience, 1:100). Samples were subsequently washed three times for 5 min each time in 10% (v/v) NGS and 0.1% (v/v) Tween 20 in PBS at room temperature with rotation. Immunofluorescence was detected by incubating samples in buffer containing either rat or rabbit APC- and PE-conjugated secondary antibodies (#A-21472, #A31556, Thermo, 1:100). After three washes of the sections as described above, nuclei were stained with DAPI (#D3571, Invitrogen). Following a final wash, sections were mounted on slides with mounting medium (#S3023, Dako) and analyzed via a confocal laser scanning microscope (LSM900, Zeiss). The number of antibody-positive cells was visually enumerated at high magnification.

### Statistical analysis

Results are presented as the mean ± standard error of the mean. Comparisons of two groups were performed via an unpaired *t*-test. Statistical significance was defined as a *p* value < 0.05. GraphPad Prism version 9.50 (GraphPad Software, San Diego, California, USA) for Windows was used to plot the data.

## Results

### GRIM-19 expression decreases in the lesional skin of bleomycin-induced SSc mice and is negatively correlated with STAT3 expression

**Fig. 1:**
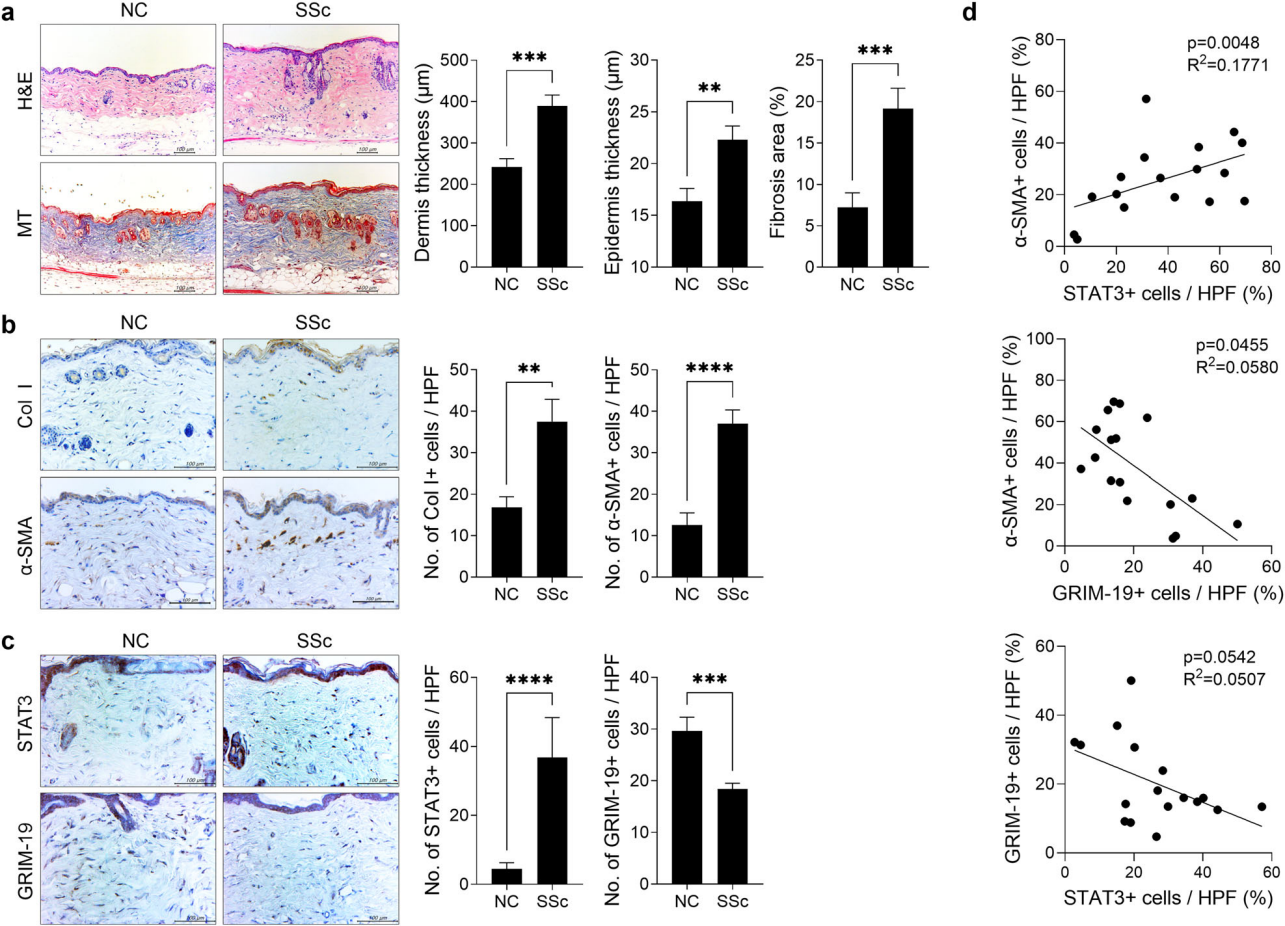
GRIM-19 levels are negatively correlated with those of STAT3 in the lesional skin of bleomycin-induced SSc mice. Eight-week-old C57BL/6 mice were subcutaneously injected (*n* = 17) or not (normal control, *n* = 17) with 50 μg of bleomycin dissolved in 100 μL of PBS daily for 2 weeks. Tissues from SSc and control mice euthanized on Day 35 after the start of bleomycin treatment were subjected to histologic analysis. **a** Skin sections stained with hematoxylin and eosin (H&E) and Masson’s trichrome (MT). The thickness of the dermis and epidermis and the percentage of fibrosis area are plotted. Original magnification: 200×; scale bar: 100 µm. **b** Skin sections stained with antibodies against collagen type 1 (Col1) and α-smooth muscle actin (SMA). The number of antibody-positive cells (mean ± SEM) is plotted. Original magnification: 400×; scale bar: 100 µm. **c** Skin sections stained with antibodies against STAT3 and GRIM-19. The number of antibody-positive cells (mean ± SEM) is plotted. Original magnification: 400×; scale bar: 100 µm. **d** The correlations between α-SMA and STAT3, α-SMA and GRIM-19, and STAT3 and GRIM-19 were analyzed on the basis of numbers of α-SMA+, STAT3+, and GRIM-19+ cells in the skin of the SSc mice shown in (**b** and **c**). **p < 0.01, ***p < 0.001, ****p < 0.0001 (unpaired nonparametric *t*-test).

H&E and Masson’s trichrome staining revealed greater dermal and epidermal skin thicknesses as well as greater collagen deposition in bleomycin-induced SSc mice than in control mice (Fig. 1a). The increased infiltration of cells that were positive for collagen type I and for α-smooth muscle actin (SMA), which is a marker of activated myofibroblasts, was also observed in the skin tissue of SSc mice (Fig. 1b). Consistent with previous reports^10,11^, the infiltration of STAT3-positive cells was detected within the lesional skin of SSc mice. In contrast, fewer GRIM-19-positive cells were detected in the lesional skin of SSc mice than in controls (Fig. 1c). In the skin of SSc mice, α-SMA and STAT3 levels were positively correlated, whereas α-SMA and GRIM-19 levels were negatively correlated. GRIM-19 and STAT3 also tended to be negatively correlated in SSc mice (Fig. 1d). These results suggest that there is a negative interaction between GRIM-19 and STAT3, which is a key regulator of fibrosis, in bleomycin-induced SSc mice.

### Overexpression of GRIM-19 reduces dermal thickness and the infiltration of STAT3-positive cells into the skin of bleomycin-induced SSc mice

**Fig. 2:**
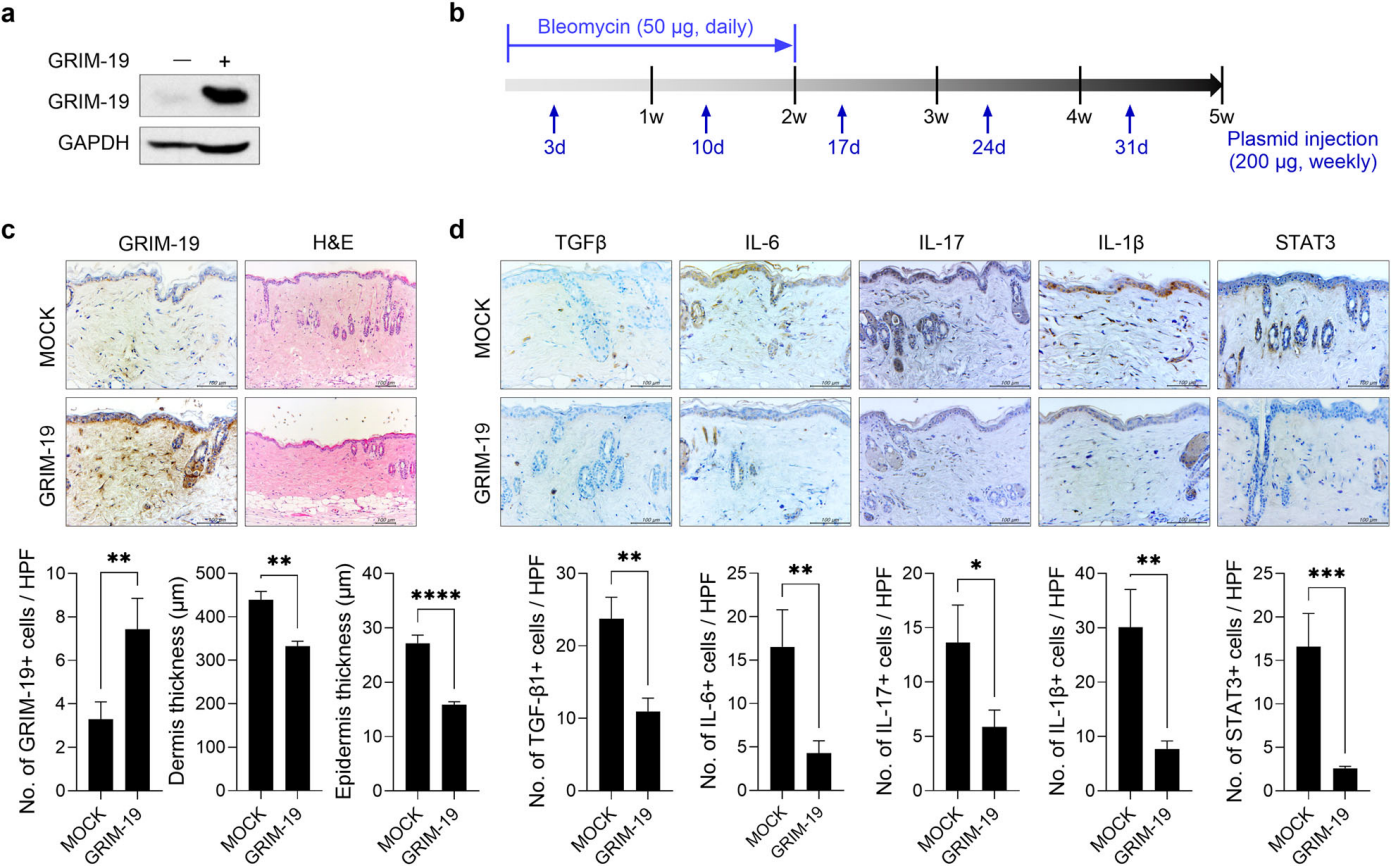
Overexpression of GRIM19 ameliorates the severity of bleomycin-induced SSc. **a** GRIM-19 protein levels were measured by Western blot in NIH3T3 cells transfected with the pFLAG-CMV-5a R12 mGRIM-19 plasmid or mock vector and cultured for 48 h. GAPDH served as the validation control. **b** A murine model of SSc was generated as described in Fig. 1. Mice were injected intravascularly with the pFLAG-CMV-5a R12 mGRIM-19 plasmid (*n* = 5) or mock (*n* = 5) vector once a week for 5 weeks from the start of bleomycin treatment. Tissues from SSc and control mice euthanized on Day 35 after the start of bleomycin treatment were subjected to histologic analysis. **c** Skin sections stained with antibodies against GRIM-19 and with H&E. The number of GRIM-19-positive cells and the thicknesses of the dermis and epidermis are plotted. Original magnification: 400×; scale bar: 100 µm. **d** Skin sections stained with antibodies against TGF-β, IL-6, IL-17, IL-1β, or STAT3. The number of antibody-positive cells (mean ± SEM) is plotted. Original magnification: 400×; scale bar: 100 µm. *p < 0.1, **p < 0.01, ***p < 0.001, ****p < 0.0001 (unpaired nonparametric *t*-test).

To determine whether the overexpression of GRIM-19 mitigates SSc, the pFLAG-CMV-5a R12 mGRIM-19 plasmid was intravenously injected once a week for 5 weeks into bleomycin-induced SSc mice, and disease severity was evaluated in comparison with that in control mice (Fig. 2b). A preliminary experiment confirmed the overexpression of the pFLAG-CMV-5a R12 mGRIM-19 plasmid in a cell line (Fig. 2a). In SSc mice, GRIM-19 overexpression increased the number of GRIM-19-positive cells in the skin. It also resulted in therapeutic effects as shown by decreases in dermal and epidermal thickness (Fig. 2c). The numbers of cells expressing TGF-β, IL-6, IL-17, or IL-1β and the infiltration of cells expressing STAT3 were also lower in SSc mice than in control mice (Fig. 2d). These results showed that GRIM-19 mitigates SSc by reducing the number of inflammatory cytokine-mediated STAT3-expressing cells in the skin.

### Overexpression of GRIM-19 reduces the frequency of Th2 and Th17 cells in bleomycin-induced SSc mice

**Fig. 3:**
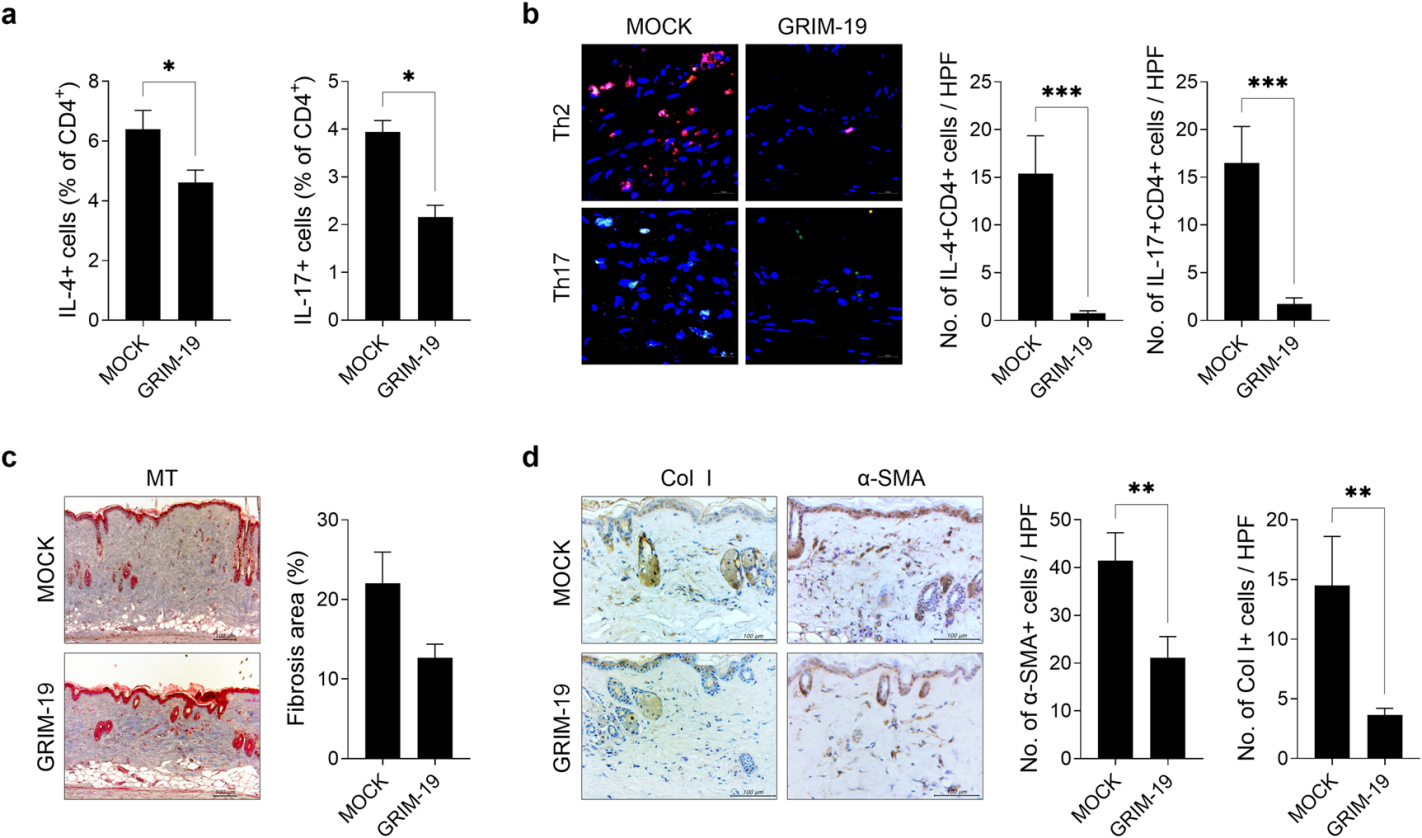
Overexpression of GRIM-19 downregulates Th2 and Th17 cells and alleviates skin fibrosis. A murine model of SSc was generated as described in Fig. 1. Mice were injected intravascularly with the pFLAG-CMV-5a R12 mGRIM-19 plasmid (*n* = 5) or mock (*n* = 5) vector once a week for 5 weeks from the start of bleomycin treatment and euthanized on Day 35. **a** The frequencies of Th2 (IL-4^+^CD4^+^) and Th17 (IL-17^+^CD4^+^) cells in the peripheral blood were analyzed via flow cytometry. **b** Skin sections were stained with fluorescently labeled antibodies against CD4 (APC), IL-4 (PE), and IL-17 (FITC), and the number of IL-4^+^CD4^+^ and IL-17^+^CD4^+^ cells was examined via confocal microscopy. **c** Skin sections stained with MT. The percentage of tissue area affected by fibrosis is plotted. **d** Skin sections were stained with antibodies against Col1 and α-SMA. The number of antibody-positive cells (mean ± SEM) is plotted. Original magnification: 400×; scale bar: 100 µm. *p < 0.1, ***p < 0.001 (unpaired nonparametric *t*-test).

To determine the effect of GRIM-19 overexpression on pathogenic T cells in SSc mice, the frequency of Th2 and Th17 cells in the peripheral blood of SSc mice injected with the pFLAG-CMV-5a R12 mGRIM-19 plasmid was analyzed via flow cytometry. Compared with control treatment, overexpression of GRIM-19 significantly decreased the numbers of Th2 and Th17 cells in SSc mice (Fig. 3a). Confocal microscopy revealed reductions in the number of Th2 and Th17 cells in the skin (Fig. 3b). These results suggest that the therapeutic effect of GRIM-19 in SSc mice is at least in part mediated by reductions in proinflammatory Th2 and Th17 cells.

### Overexpression of GRIM-19 attenuates skin fibrosis in bleomycin-induced SSc mice

The effect of GRIM-19 overexpression on SSc-associated fibrosis was assessed in skin sections stained with MT via immunohistochemistry. GRIM-19 overexpression reduced excessive collagen deposition in the lesional skin of control mice, while immunohistochemistry revealed fewer collagen type I-positive cells and α-SMA-positive cells (Fig. 3c, d). These results demonstrate the ability of GRIM-19 to alleviate fibrosis in the lesional skin of SSc mice.

### Mitophagic dysfunction promotes the development of bleomycin-induced SSc

**Fig. 4:**
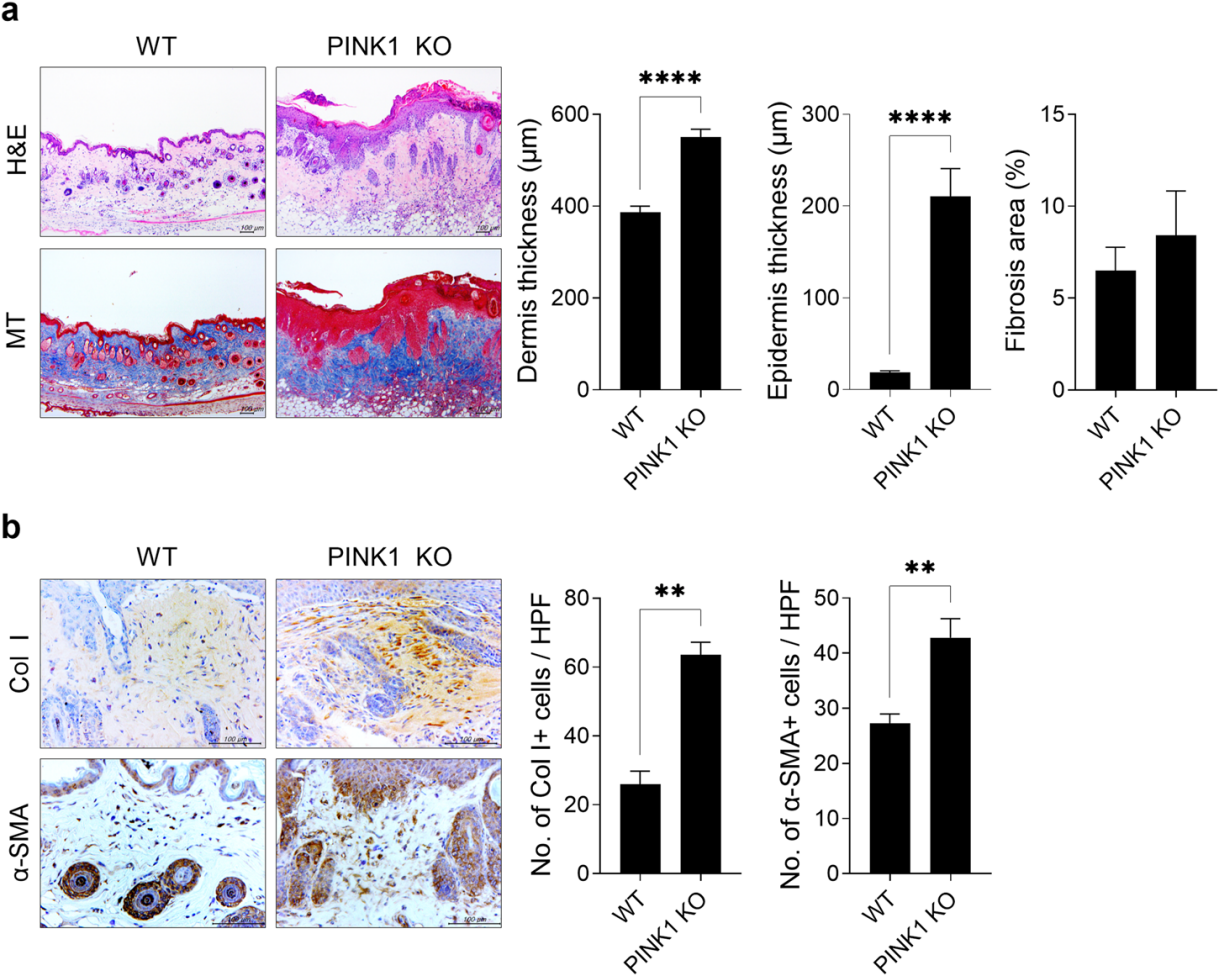
PINK1 deletion-induced mitophagy impairment promotes the progression of bleomycin-induced SSc. The SSc model was induced by subcutaneously injecting 50 μg of bleomycin dissolved in 100 μL of PBS daily for 2 weeks into 8-week-old mice lacking PINK1 and wild-type mice (*n* = 3/group). Tissues from SSc and control mice euthanized on Day 35 after the start of bleomycin treatment were subjected to histologic analysis. **a** Skin sections were stained with H&E and MT. The thickness of the dermis and epidermis and the percentage of tissue area involved in fibrosis are plotted. Original magnification: 100×; scale bar: 100 µm. **b** Skin sections stained with antibodies against Col1 and α-SMA. The number of antibody-positive cells (mean ± SEM) is plotted. Original magnification: 400×. Scale bar: 100 µm. **p < 0.01, ****p < 0.0001 (unpaired nonparametric *t*-test).

GRIM-19 directly binds to STAT3 to mediate its mitochondrial translocation^17,21^. In turn, mitoSTAT3 engages in the autophagic removal of damaged mitochondria, which is a process called mitophagy^23^. Thus, the effect of mitophagy on fibrosis development was examined to determine whether the GRIM-19-mediated increase in mitoSTAT3 affects the development of SSc by promoting mitophagy. Bleomycin-mediated SSc development was therefore evaluated in mice deficient in PINK1, which is a mitophagy inducer^24^. Compared with that in control mice, the suppression of mitophagy due to PINK1 deletion significantly increased dermal and epidermal thickness in SSc mice (Fig. 4a). Owing to the dramatic increase in epidermal thickness in PINK1 knockout mice with bleomycin-induced SSc, no significant increase in the area of collagen deposition was observed via MT staining. However, the infiltration of cells that were positive for collagen type I and α-SMA was significantly greater in those mice than in control mice (Fig. 4b). These results indicate that mitophagy dysfunction can promote SSc development and fibrosis progression.

### GRIM-19-mediated induction of mitophagy through mitoSTAT3 alleviates fibrosis progression in vitro

**Fig. 5:**
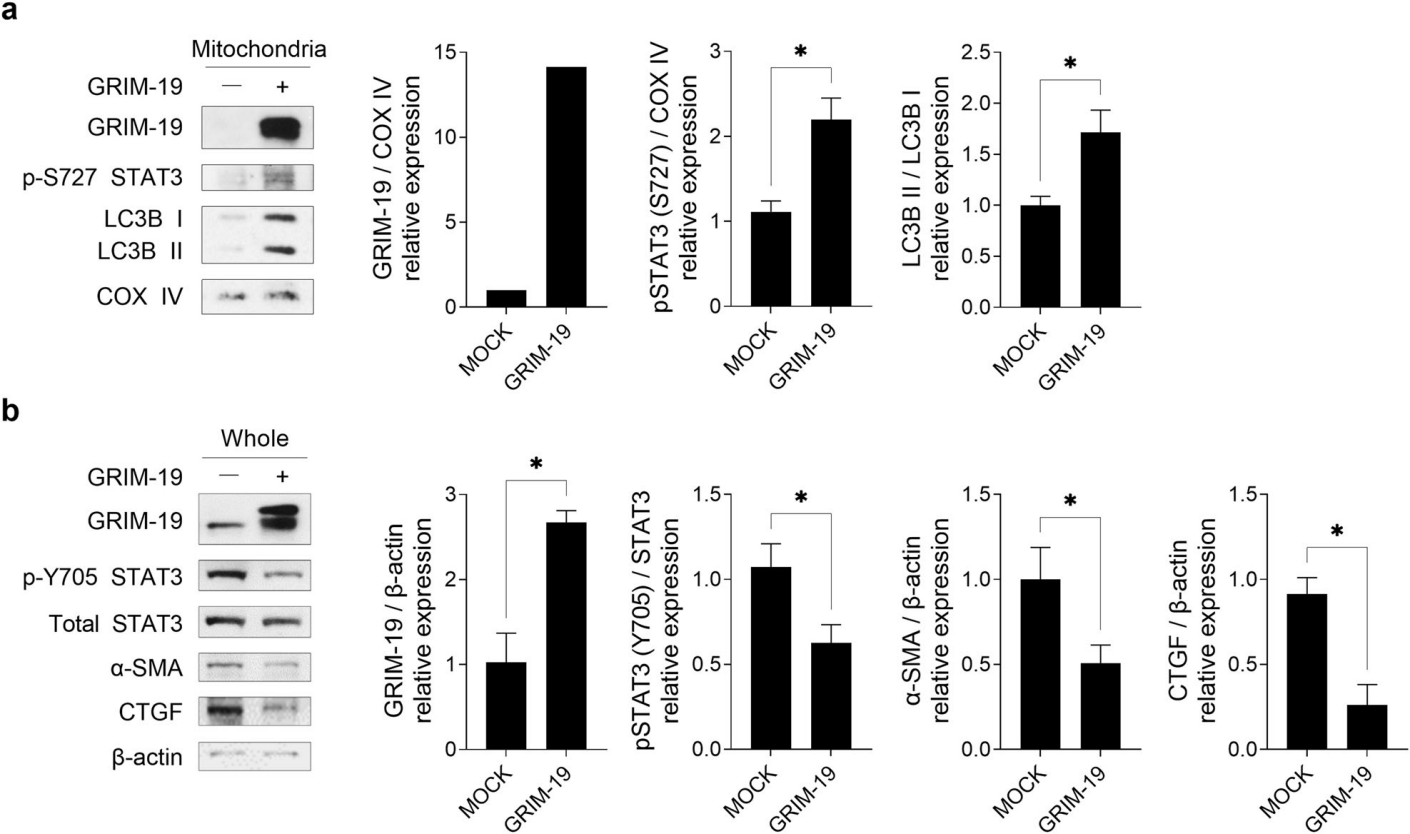
GRIM-19 promotes mitophagy via increased mitochondrial STAT3 and alleviates fibrosis. HEK293 cells were transfected with empty vector or the pcDNA3.0 hGRIM-19 plasmid and then serum-starved for 16–18 h. **a** The mitochondrial fraction was isolated from cells treated with IL-6 (10 ng/mL) for 12 h, and levels of GRIM-19 and STAT3 phosphorylated at S727 and LC3B were examined via Western blotting. COX IV served as the validation control for the mitochondrial fractions. The ratio of the relative density of the target protein normalized to that of each validation control (mean ± SEM) is plotted. **b** Cells were treated with IL-6 (10 ng/mL) for 48 h, and the protein levels of GRIM-19, STAT3 phosphorylated at Y705, total STAT3, α-SMA and CTGF proteins in whole-cell lysates were examined via Western blotting. β-actin served as the validation control. The ratio of the relative density of the target protein normalized to each validation control (mean ± SEM) is plotted. *p < 0.05 (unpaired nonparametric *t*-test).

The effect of a GRIM-19-mediated increase in mitoSTAT3 on mitophagy was first determined in vitro. HEK293 cells were transfected with the GRIM-19 plasmid, and the conversion of LC3B I to LC3B II in the mitochondrial fraction was examined by Western blotting. The overexpression of GRIM-19 increased the level of STAT3 phosphorylation at S727 and the LC3B-II/LC3B-I ratio in mitochondria, which indicated that a GRIM-19-mediated increase in mitoSTAT3 accelerated mitophagy (Fig. 5a). The effect of the GRIM-19-mediated increase in mitoSTAT3 on fibrosis was investigated by stimulating IL-6 expression in HEK293 cells overexpressing GRIM-19 to promote a STAT3-dependent increase in fibrosis^25^. Both the level of STAT3 phosphorylation at Y705 and levels of fibrosis mediators α-SMA and CTGF were reduced (Fig. 5b)^26^.

These results suggest that mitoSTAT3, and thus GRIM-19, plays a role in alleviating fibrosis by inducing mitophagy.

### Mitochondrial STAT3 ameliorates the progression of bleomycin-induced SSc

**Fig. 6:**
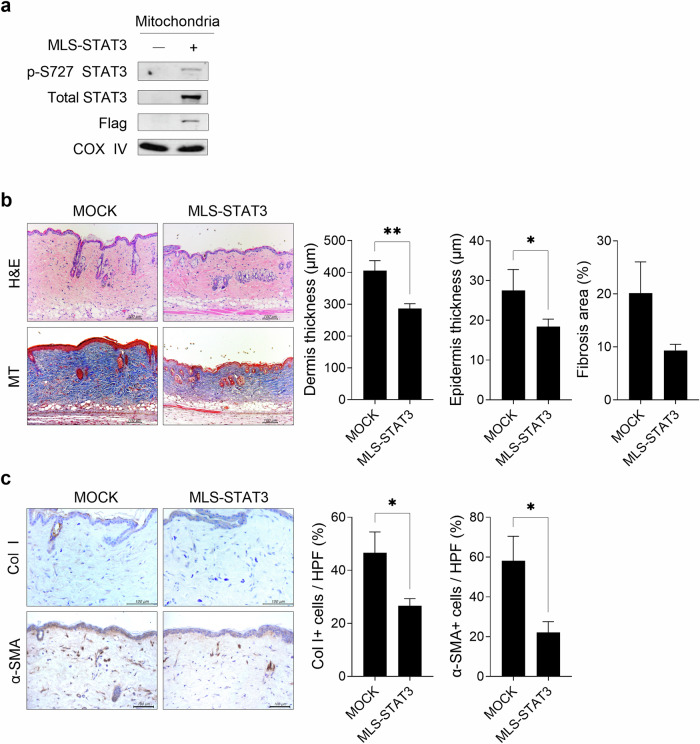
Mitochondrial STAT3 increases the severity of bleomycin-induced SSc. **a** NIH3T3 cells were transfected with the pEG-MLS-mSTAT3-FLAG or mock vector, and mitochondrial fractions were isolated after 48 h. The levels of Ser727-phosphorylated STAT3 and total STAT3 were assessed via Western blotting. COX IV served as the validation control. **b**, **c** A murine model of SSc was generated as described in Fig. 1. The mice were injected intravascularly with the MLS-STAT3 (*n* = 5) or mock (*n* = 5) vector once a week for 5 weeks from the start of bleomycin treatment. Tissues from SSc and control mice euthanized on Day 35 after the start of bleomycin treatment were subjected to histologic analysis. **b** Skin sections were stained with H&E and MT. The thickness of the dermis and epidermis and the percentage of tissue area involved in fibrosis are plotted. Original magnification: 200×; scale bar: 100 µm. **c** Skin sections were stained with antibodies against Col1 and α-SMA. The number of antibody-positive cells (mean ± SEM) is plotted. Original magnification: 400×; scale bar: 100 µm. *p < 0.05, **p < 0.01 (unpaired nonparametric *t*-test).

To determine whether mitoSTAT3 plays a role in alleviating the development of SSc, MLS-STAT3 was intravascularly injected into bleomycin-induced SSc mice once a week for 5 weeks. A preliminary experiment confirmed the overexpression of MLS-STAT3 in mitochondrial fractions isolated from NIH3T3 cells (Fig. 6a). In bleomycin-induced SSc mice, MLS-STAT3 overexpression significantly decreased dermal and epidermal thickness with a reduction trend in areas of collagen deposition (Fig. 6b). MLS-STAT3 overexpression also reduced the infiltration of cells that were positive for collagen type-I and α-SMA into the lesional skin of SSc mice compared with that of controls (Fig. 6c). These results suggest a role for GRIM-19-mediated mitoSTAT3 in hindering the development of bleomycin-induced SSc in mice.

## Discussion

GRIM-19 reduces fibrosis by promoting mitophagy (via the induction of mitoSTAT3) during SSc development in bleomycin-treated mice. Levels of fibrotic factors were positively correlated and levels of GRIM-19 were negatively correlated with those of STAT3. Our GRIM-19 gene therapeutic approach in SSc mice reduced dermal thickness and the extent of skin invasion by cells producing TGF-β, IL-6, IL-17, IL-1β, and STAT3. Consistent with the findings of previous studies on inflammation^18,19^, GRIM-19 overexpression decreased the numbers of Th2 and Th17 cells in the peripheral blood and spleen of SSc mice in addition to inhibiting the skin invasion of cells expressing fibrotic markers. As GRIM-19 binds directly to STAT3^17^ and given the previously described fibrotic effects of STAT3^11^, our results indicate that the mechanism underlying the observed GRIM-19-mediated amelioration of SSc involves the control of nuclear STAT3 transcription.

GRIM-19, also termed NADH:ubiquinone oxidoreductase subunit A13, is a component of mitochondrial complex I of the ETC^27^ and plays major roles in the nucleus. Among the reported functions of GRIM-19 are reductions in oxidative phosphorylation, ATP production, and oxygen consumption and a delay in cell growth^27^. GRIM-19 also mediates the mitochondrial translocation of Ser727-phosphorylated STAT3 and enhances the function of ETC complex I^28–30^.

Several recent studies have reported that metabolic syndrome caused by mitochondrial dysfunction promotes diabetes and obesity^31^. Radiation-induced skin fibrosis is attributable to mutations in the gene encoding thioredoxin reductase 2, which is involved in mitochondrial oxygen-radical scavenging^32^. A BH3 mimetic drug that inhibits the antiapoptotic protein Bcl-XL was shown to ameliorate bleomycin-induced SSc via mitochondrial regulation^33^. Thus, despite few studies examining the role played by mitochondria in SSc, the above findings suggest a relationship. Mitophagy, the selective autophagic elimination of aged and damaged mitochondria, is critical for the maintenance of cellular homeostasis under both normal and stress conditions^34,35^. Previous studies revealed an association between Ser-727 STAT3 phosphorylation and *Helicobacter pylori*-induced autophagic removal of damaged mitochondria, and that *H. pylori*-induced mitochondrial translocation of phosphorylated STAT3 is mediated by GRIM-19^36^. Conversely, mitochondrial complex I plays an important role in mitophagy^23^. Rapamycin reduces fibrosis by inducing autophagy in an animal model of SSc^37^; however, the inhibition of autophagy also reduces fibrosis^38,39^.

We investigated the relationship between mitophagy and SSc by exploring whether GRIM-19 inhibits nuclear STAT3-mediated fibrosis and thus ameliorates SSc by influencing mitoSTAT3 levels and ultimately mitophagy. In a previous study, SSc was induced in mice deficient in PINK1, which is the master regulator of mitophagy; the resulting increase in mitophagy was associated with SSc amelioration and suggests a key role of mitophagy in fibrosis development^24^. The overexpression of GRIM-19 was also shown to promote mitophagy by increasing the expression of mitoSTAT3, which is accompanied by a decrease in the levels of fibrotic markers. This is the first study to show that GRIM-19 reduces fibrosis by enhancing mitophagy via mitoSTAT3 induction. MLS-STAT3 gene therapy in mice with SSc reduced dermal thickness and resulted in antifibrotic effects. Thus, the GRIM-19-mediated increase in mitoSTAT3 may aid SSc patients.

JAK is a nonreceptor tyrosine kinase that plays an important role in cytokine and growth factor signaling. When ligands, such as cytokines, bind to their receptor, the JAK kinase is activated and JAK‒STAT signal transduction is initiated. The IL-6/JAK/STAT signaling pathway is involved in the pathogenesis of various inflammatory and autoimmune diseases, and JAK inhibitors are emerging as promising new therapeutics for the treatment of autoimmune diseases^40^. The injection of tofacitinib, a synthetic kinase inhibitor that targets the activity of JAK1 and JAK3, into bleomycin-induced SSc mice attenuated disease progression^41^. In addition, the efficacy of JAK inhibitors has been reported in patients with SSc^42^. GRIM-19 targets STAT3, which is downstream of JAK, and in this study, we demonstrated that strengthening GRIM-19 inhibits the activity of STAT3. Future studies on the mechanism of action by which GRIM-19 targets STAT3 may contribute to the development of more pathogenesis-selective therapeutics.

In summary, our experiments revealed that functional and morphological changes in mitochondria are enhanced by increasing mitoSTAT3 levels during mitophagy regardless of the PINK1 status. Overall, mitochondrial dysfunction worsened SSc, whereas the removal of damaged mitochondria via mitophagy slowed disease progression.

## Data Availability

All data generated or analyzed during this study are included in the published article.
